# The Utility of Point of Care Test for Soluble ST2 in Predicting Adverse Cardiac Events during Acute Care of ST-Segment Elevation Myocardial Infarction

**DOI:** 10.1155/2018/3048941

**Published:** 2018-06-26

**Authors:** Anggoro Budi Hartopo, Indah Sukmasari, Ira Puspitawati

**Affiliations:** ^1^Department of Cardiology and Vascular Medicine, Faculty of Medicine, Public Health and Nursing, Universitas Gadjah Mada and Dr. Sardjito Hospital, Yogyakarta, Indonesia; ^2^Department of Clinical Pathology, Faculty of Medicine, Public Health and Nursing, Universitas Gadjah Mada and Dr. Sardjito Hospital, Yogyakarta, Indonesia

## Abstract

**Introduction:**

Soluble ST2 (sST2) is increased during acute myocardial infarction. The point of care test (POCT) for sST2 is currently available. The aim of this study was to investigate the utility of the sST2 POCT measurement for predicting adverse cardiac events during acute care of ST-elevation myocardial infarction (STEMI).

**Patients and Methods:**

This research used a cohort study design. Consecutive patients with STEMI were enrolled. Soluble ST2 level was measured from peripheral blood taken on admission with POCT. Observation during acute intensive care was conducted to record adverse cardiac events. Two groups were assigned based on median sST2 level, that is, supramedian and inframedian group. The incidence of adverse cardiac events between groups was analyzed. A *p* value < 0.05 was statistically significant.

**Results:**

We analyzed 95 subjects with STEMI and 10 patients with stable coronary artery disease as controls. The median sST2 level was significantly higher in subjects with STEMI as compared to controls (152.1 ng/mL versus 28.5 ng/mL, *p* < 0.01). Among subjects with STEMI, the supramedian group had higher incidence of adverse cardiac events than the inframedian group (38.3% versus 12.5%, *p*=0.004). Multivariable analysis showed that supramedian sST2 level was independently associated with increased incidence of adverse cardiac events (adjusted OR 6.27; 95% CI: 1.33–29.47, *p*=0.020).

**Conclusions:**

The sST2 POCT measurement was useful to independently predict adverse cardiac events during acute intensive care of STEMI.

## 1. Introduction

Acute myocardial infarction with ST-segment elevation (STEMI) is a clinical syndrome which reflects the transmural necrosis of myocardia due to occlusive thrombus in the coronary artery segment. The prevalence of STEMI ranges from 25% to 45% among patients with acute myocardial infarction [1]. The management of STEMI with reperfusion strategies, both fibrinolysis and primary coronary intervention, and pharmacology therapy have successfully reduced the major adverse cardiac events and mortality [1]. However, in many high-risk patients, the mortality and major adverse cardiac events rate are persistently high [2].

Several independent predictors for major adverse cardiac events following STEMI have been identified such as age, Killip class on admission, delay of reperfusion time, cardiac arrest, tachycardia, hypotension, anterior infarct location, previous myocardial infarction, diabetes mellitus, smoking, renal function, and increased biomarkers for myocardial necrosis [3]. Until recently, the role of biomarkers as a predictor for major adverse cardiac events and mortality in acute hospital care has not been well established. The biomarkers of myocardial necrosis, such as creatine kinase-MB (CK-MB) and troponin, have been included in the Grace scoring system which estimates the risk for mortality and nonfatal major adverse cardiac events during hospitalization among acute coronary syndrome patients. However, it is not considered specifically useful for STEMI [3].

Novel biomarkers have been identified and investigated in their role specifically in acute myocardial infarction. Soluble ST2 (sST2), which is released during myocardial stretching, is one promising biomarker [4]. In patients with STEMI, in which transmural myocardial injury occurred, myocardia in the left ventricle undergoes additional stretching. This process involves the release of biomarkers such as NT-proBNP and sST2 by myocardia and cardiac fibroblasts [4]. In the initial phase, during which clinical signs and symptoms of left ventricle dysfunction have not yet appeared, the release of sST2 may give an indication of ongoing left ventricle stretching and predict the development of left ventricle dysfunction.

Several studies have concluded that increased sST2 in the initial phase of STEMI is associated with adverse cardiac effect, both in the short term and long term. However, currently no guideline recommends the examination of sST2 as a predictor biomarker to guide treatment for STEMI. Therefore, confirmatory research should be performed to provide more solid evidence of sST2 implication in predicting major adverse cardiac events in patients hospitalized with STEMI. Furthermore, the current availability of the sST2 point of care test (POCT), which allows the result to be known more quickly, is suitable in the acute clinical setting such as STEMI. This study aimed to investigate the utility of the sST2 POCT measurement in predicting adverse cardiac events in patients hospitalized with STEMI.

## 2. Methods

### 2.1. Research Design

The research used a cohort study design. The subjects were patients with STEMI admitted to the Intensive Cardiac Care Unit (ICCU) of Dr. Sardjito Hospital, Yogyakarta, Indonesia. The subjects were enrolled from the emergency unit of Dr. Sardjito Hospital, Yogyakarta, Indonesia, where they were stabilized and subsequently admitted to ICCU. The subjects' enrollment (from April 2014 to January 2015) was conducted by consecutive sampling. The diagnosis of STEMI was determined based on international guidelines, that is, angina symptoms, electrocardiogram criteria, and elevated cardiac enzyme markers [5]. The observations of subjects were conducted during intensive cardiac care in the ICCU.

### 2.2. Research Subjects

We included the patients diagnosed with STEMI, both male and female patients, with age between 35 and 75 years old, the onset of anginal pain ≤24 hours, and patients without previous fibrinolytic or heparin treatment before reaching our hospital emergency unit. We excluded the patients with previously known chronic heart failure (NYHA class ≥ II), chronic kidney disease stage IV-V, hepatic cirrhosis, chronic inflammatory diseases (such as chronic arthritis, psoriasis, and inflammatory bowel disease), malignancy, and patients with concurrent acute infection, sepsis, and acute stroke during observation. The subjects competed and signed an informed consent form to participate in the research. As controls, we recruited patients with stable coronary artery disease (SCAD) who came to our hospital for coronary angiography and PCI. These patients were assigned as controls in this study. The study was approved by the ethics committee of the Faculty of Medicine, Public Health and Nursing, Universitas Gadjah Mada, Yogyakarta, Indonesia.

### 2.3. Laboratory Examination

For laboratory examination, blood samples were drawn from antecubital veins in a supine position on hospital admission before any procedures were performed. The blood was then centrifuged and inserted into an automated blood cell counter for hematology examination and chemical analyzer for blood chemistry examination. Cardiac biomarkers were measured as routine procedure, that is, CK-MB by the immunological UV assay method with Cobas c501 (Roche) and troponin I by the enzyme-linked fluorescent assay (ELFA) method with VIDAS (Biomerieux) in the central hospital laboratory. In controls, blood samples were drawn from antecubital veins in a supine position on hospital admission before coronary angiography and PCI were performed.

From the blood sample, an aliquot of serum was collected and saved in −80°C freezer until further analysis for sST2 examination. Soluble ST2 level was measured from frozen samples. The aliquot was thawed, left in room temperature, and used for sST2 quantification with ASPECT-PLUS ST2 rapid test (Critical Diagnostics, San Diego, CA, USA). The calculation of sST2 was performed based on the manufacturer's manual instructions and previous report [6]. Briefly, the ASPECT-PLUS ST2 test cassette was warmed under room temperature for 15 minutes, the foil pouch was opened, and 30–40 microliter serum sample was dropped into the sample well. An amount of 2 drops of test buffer was placed into the test buffer well. The ASPECT-PLUS ST2 test cassette was inserted into the ASPECT reader (Critical Diagnostics, San Diego, CA, USA). The reader processed the measurement (±20 minutes), and the quantitative sST2 levels (ng/mL) were exhibited on the display. We recorded the result of sST2 measurement in print. Based on a measurement limit, serum sST2 levels <12.5 ng/mL or >250 ng/mL were reported by the ASPECT Reader as <12.5 ng/mL and >250 ng/mL value [6].

### 2.4. Observation and Outcome

The initial treatments of the subjects with STEMI in the emergency unit were double antiplatelet, revascularization procedure, that is, primary percutaneous coronary intervention (PCI) or fibrinolysis and anticoagulant with heparin. Time for revascularization was determined as time (in minutes) from medical contact (i.e., emergency admission in our hospital) to start of reperfusion therapy by intravenous injection of fibrinolytic agents and time to balloon inflation in subjects treated with fibrinolysis and primary PCI, respectively. The initial management was performed by attending cardiologists according to patients' clinical condition and severity. After initial treatments, the subjects were transferred to the ICCU and managed according to the discretion of the attending cardiologists.

Transthoracic echocardiography (TTE) was performed on all subjects with STEMI within 48 hours of admission in the ICCU. The GE Vivid S6 (GE Healthcare) echocardiography machine was used for TTE procedure. Left ventricle (LV) ejection fraction was obtained using the modified Simpson's method on the standard simultaneous apical four-chamber and two-chamber views. The interpretation of TTE results was performed by experienced cardiologists.

The observation of subjects with STEMI was conducted during acute intensive care in the ICCU until discharge from the ICCU or fatal event occurred. The adverse cardiac events were a composite of cardiac death, acute heart failure, cardiogenic shock, reinfarction, and resuscitated ventricular arrhythmia (VT/VF). The adverse cardiac events were assessed and managed by attending cardiologists unaware of the sST2 measurement. A cardiac death was mortality by cardiac causes. Acute heart failure was the clinical symptoms and signs of congestion and the use of intravenous diuretics. Cardiogenic shock was systolic blood pressure <90 mmHg with symptoms and signs of low perfusion and the use of inotropics or/and vasopressor drugs. Reinfarction was newly developed continuous anginal pain, ST-elevation electrocardiogram and increased CK-MB or troponin I in previously stable patients. Resuscitated VT/VF was the return of spontaneous circulation following cardiopulmonary resuscitation and defibrillation after the episode of VT/VF.

### 2.5. Statistical Analysis

The median sST2 level between subjects with STEMI and controls was compared with the Mann–Whitney *U* test. Based on sST2 levels, the subjects with STEMI were divided into two groups, that is, supramedian group and inframedian group. The cutoff point to divide the groups was the median value. The incidence of adverse cardiac events between groups was compared and analyzed with the chi-squared test. The median sST2 level among subjects with STEMI based on occurrence of adverse cardiac events, and the control was compared with the Kruskal–Wallis test. The Student's *t*-test was performed to compare continuous variables with normal data distribution. Univariate and multivariable analyses were performed to determine the predictors for adverse cardiac events. The logistic regression test was performed as a multivariable analysis to evaluate whether sST2 levels independently predict adverse cardiac events. A statistically significant result was determined by *p* value < 0.05.

## 3. Results

The subjects with STEMI for this research were 97 patients. The controls were 10 patients. The sST2 measurement was performed by the ASPECT-PLUS ST2 POCT cassette and ASPECT reader with the detection range of 12.5 ng/mL–250 ng/mL. Two subjects with STEMI were excluded from the analysis because the ASPECT-PLUS ST2 POCT result strip was undetermined. Therefore, 95 subjects with STEMI were analyzed. The median sST2 level was significantly increased in subjects with STEMI as compared to controls (152.1 ng/mL versus 28.5 ng/mL, with *p* value < 0.01, resp.). Subjects with STEMI had significantly increased leukocyte count and blood glucose level as compared to controls. The characteristics and sST2 level between subjects with STEMI and controls are compared and shown in Table 1.

**Figure 1:**
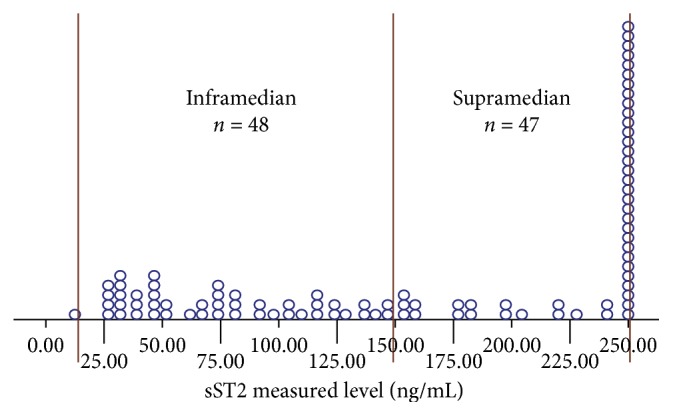
The distribution of the sST2 measured level (ng/mL) and median of 152.1 ng/mL as a cutoff point in subjects with STEMI. As many as 32 subjects have the sST2 level more than the upper measured limit (i.e., >250 ng/mL), and 1 subject has sST2 level less than the lower measured limit (i.e., <12.5 ng/mL).

In subjects with STEMI, as many as 32 subjects (33.7%) had sST2 level more than the upper measured limit (i.e., >250 ng/mL) and 1 subject had sST2 level less than the lower measured limit (i.e., <12.5 ng/mL). We determined the level of 250 ng/mL for 32 subjects with value beyond the upper measured limit and 12.5 ng/mL for 1 subject with value below the lower measured limit. The median value of sST2 was 152.1 ng/mL. The supramedian group consisted of 47 subjects and inframedian group consisted of 48 subjects. The distribution of sST2 values is depicted in Figure 1.

Subjects with supramedian sST2 levels and those with inframedian sST2 had no significant difference in demography, cardiovascular risk factors, admission clinical picture, most of initial laboratory examination, infarct location, and initial treatment strategy. Patients in the supramedian group had significantly higher troponin I and CK-MB levels than the inframedian group, indicating increasing myocardial necrosis. The initial treatments of the subjects did not significantly differ between groups. The comparison of characteristics between the supramedian and inframedian groups is shown in Table 2.

The proportion of initial revascularization procedure, that is, primary PCI or fibrinolysis, was comparable between groups. In subjects who underwent primary PCI, the time to revascularization was not significantly different between groups. This also applied to the time to revascularization for fibrinolysis. The rate of failed fibrinolysis and subsequent rescue PCI did not significantly differ between groups either.  Table 3 shows the mode of revascularization and time to revascularization between supramedian and inframedian groups.

**Figure 2:**
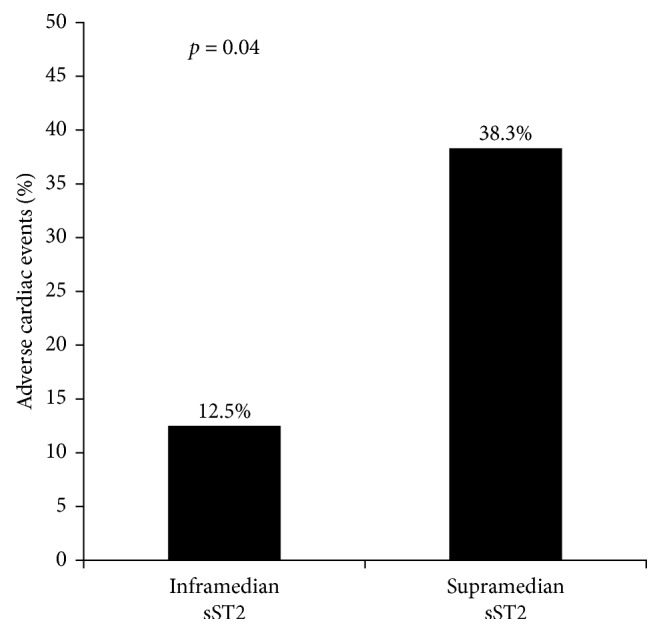
The incidence of adverse cardiac events between inframedian and supramedian sST2 groups. The adverse cardiac events were significantly higher (38.3%) in the supramedian group as compared to the inframedian group (12.5%); *p* value for the difference was 0.04 (chi-squared test).

**Figure 3:**
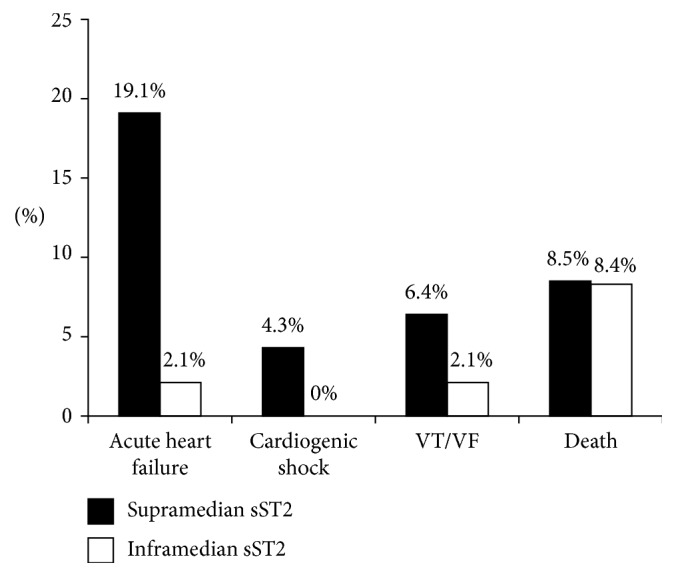
The incidence of individual adverse cardiac events between inframedian and supramedian sST2 groups. The most common adverse cardiac event was acute heart failure. The mortality rate was similar between the supramedian and inframedian sST2 groups.

**Figure 4:**
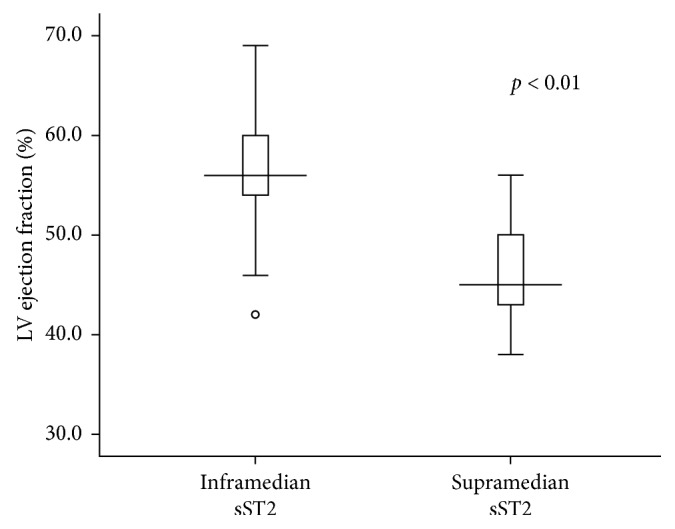
The boxplot of left ventricle (LV) ejection fraction between inframedian and supramedian sST2 groups. In the supramedian sST2 group, there was significantly reduced LV ejection fraction indicating worse LV systolic dysfunction (Student's *t*-test, *p* value < 0.01).

The incidence of adverse cardiac events was significantly higher in the supramedian group as compared to those in the inframedian group (38.3% versus 12.5%, *p* value = 0.04, resp., as shown in Figure 2). Among adverse cardiac events, acute heart failure was the most frequently occurring event in the supramedian group (19.1% versus 2.1%, resp.), whereas mortality was similar between groups.  Figure 3 shows the incidence of individual adverse cardiac events between the supramedian and inframedian groups. Furthermore, the supramedian group had significantly reduced LV ejection fraction measured during intensive hospitalization as compared to the inframedian group (46.4 ± 4.7% versus 56.9 ± 6.0%, *p* value < 0.01), indicating reduced LV systolic function in subjects with higher sST2 level during hospitalization (as shown in Figure 4).

**Figure 5:**
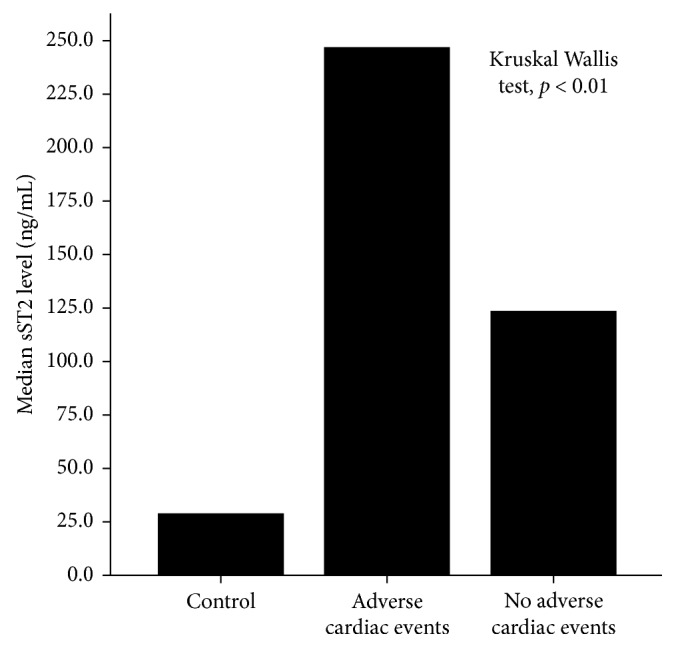
The median sST2 level was significantly the highest in STEMI subjects with adverse cardiac events, followed by STEMI subjects with no adverse cardiac events. The controls had the least median sST2 level.

The median value of sST2 level was significantly highest in subjects with STEMI-experienced adverse cardiac events (246.6 ng/mL), followed by subjects with STEMI with no adverse cardiac events (122.9 ng/mL), and the lowest was in the control (28.5 ng/mL); the *p* value of difference was <0.01 (as shown in Figure 5).

A univariate analysis showed that supramedian sST2 was significantly associated with increased incidence of adverse cardiac events, with OR 4.35 (95% CI: 1.54–12.27, *p* value 0.006). Other variables which were associated with increased incidence of adverse cardiac events are depicted in Table 4. These variables were interacted in the multivariable analysis using logistic regression tests.

A stepwise multiple logistic regression analysis showed that supramedian sST2 level was an independent predictor of increased incidence of adverse cardiac events. The adjusted OR was 6.27 (95% CI: 1.33–29.47, *p* value 0.020). Another significant variable which emerged as an independent predictor along with supramedian sST2 was older age (as shown in Table 4).

## 4. Discussion

Our study indicates that, on admission, the sST2 POCT measurement in the acute intensive care of STEMI is useful to predict adverse cardiac events during intensive hospitalization. Using median value for cutoff point, the sST2 value above the median is predictive for short-term adverse cardiac events. However, since the range of sST2 value in the POCT system is limited, one third of patients had sST2 level out of the detectable range, mostly beyond it. The simplistic and rapidity nature of the POCT system is beneficial in the acute care setting, where the quick laboratory result is mandatory to guide clinical decisions and treatment strategy. In the context of STEMI, the use of POCT in the biomarker measurement is greatly beneficial to predict adverse cardiac events.

Suppression of tumorigenicity 2 or ST2 is a receptor protein which is initially recognized as an orphan protein involved in the regulation of inflammation [4]. The ST2 protein is a member of the Interleukin-1 (IL-1) family, which shares similarity in terms of intracellular domain structure, that is, Toll/IL-1 receptor [4, 7]. This protein receptor has the role as a transmembrane receptor for several interleukins and regulates leukocyte response to cytokine activation [7]. In the systemic inflammation and/or infection, sST2 is significantly increasing and predictive for worse outcome [8, 9]. Acute myocardial infarction is hallmarked by mobilization of inflammatory cytokines and leukocytes due to ongoing necrotic tissue, the condition which stimulates the release of sST2. In our study, both mean leukocyte counts and sST2 level are above the normal values; however, leukocytosis is not significantly associated with the supramedian sST2 level.

The expression of ST2 protein is significant in myocardia [10]. Among genes activated in the myocardial that are undergoing mechanical stretching, an ST2 gene is one of the most stimulated [10]. In the mechanically stretched myocardium, alternative promoters in the ST2 gene will be activated and form a stunted shape of ST2, dubbed sST2, which can be detected in the blood circulation [11]. In the myocardia and cardiac fibroblasts, activation of the promoter region results in the majority of sST2 production and the minority of transmembrane ST2 transcripts [10, 11]. In acute myocardial infarction, necrotic and penumbra parts of left ventricular myocardia diminish their mobile and stretch capability. Therefore, the intraventricular pressure is delivered among healthy myocardia which endure additional wall tension. As a reaction to increased left ventricular wall tension and augmented intraventricular pressure, cardiomyocytes expand and release several factors which function to increase the myocardial performance [12]. Under this mechanical stretching, in the context of STEMI, the ST2 gene is activated and sST2 protein is released in the circulation [10].

The circulating sST2 acts as a decoy receptor for IL-33, which is attached to the transmembrane form of ST2 and conditions myocardial adaptation from mechanical strain [4, 10]. The ligation of IL-33 with sST2 in the circulation reduces attachment capacity of IL-33 to myocardial transmembrane ST2, hence lessening the protection of cardiomyocytes mediated by IL-33-ST2 ligation [10]. The ligation of IL-33 with transmembrane ST2 is cardioprotective to salvage cardiomyocytes from apoptosis due to strained left ventricular dimensions [13, 14]. The loss of protective effects, due to increased circulating sST2 and consequently higher decoy IL-33 ligation, puts left ventricular (LV) function at risk. Our study shows that the supramedian level of sST2 is associated with cardiac adverse events during intensive care which is mostly due to LV dysfunction. The admission Killip class, an indication of LV disturbance due to myocardial infarction, tended to be higher in patients with supramedian sST2. Furthermore, our results showed that subjects with supramedian sST2 also had reduced LV ejection fraction, indicating LV systolic dysfunction.

Our study shows that sST2 POCT measurement on admission is useful to identify patients with higher risk to develop adverse cardiac events during intensive care. The risk is about sixfold higher in those with sST2 supramedian levels on admission. The risk has been predicted early in the admission phase before the patients finally develop overt clinical events during acute intensive care, mostly due to left ventricular dysfunction. Currently, circulating sST2 has not been approved in the clinical guideline as a prognostic biomarker of adverse outcomes in acute myocardial infarction. In the guideline of heart failure, sST2 determination is useful as a biomarker for additive risk stratification, especially in the acute phase [15]. However, at present, there is still a lack of definite evidence for the utility of sST2 measurement in heart failure management practice [16].

Our study findings corroborated other clinical trials that have concluded the role of sST2 in predicting major adverse cardiovascular events following the episode of STEMI. Shimpo et al. investigated the impact of circulating sST2 in STEMI patients and found that sST2 highest quintiles associated with increased major adverse cardiovascular events during intensive hospitalization and 30 days after hospitalization [17]. Sabatine et al. found that supramedian values of circulating sST2 predicted in-hospital death and subsequent adverse cardiovascular events in STEMI patients undergoing fibrinolytic therapy [18].

In STEMI, primary PCI and fibrinolysis are recommended as modalities of revascularization. Our hospital is a PCI-capable hospital which has routinely performed primary PCI for STEMI patients. However, due to time delays in performing primary PCI, fibrinolytic treatment is still more often performed in our hospital. In this study, the proportion of primary PCI or fibrinolysis was not significantly different between groups, although in the supramedian group, the proportion of fibrinolysis tended to be higher. Additionally, the time to revascularization was not significantly different between groups, both in primary PCI and fibrinolysis.

In-hospital course of STEMI patients worsened in those with sST2 value >35 ng/mL, that is, the cutoff value of increased sST2 level in heart failure [19]. Other investigators have concluded similar findings with longer observation periods [20, 21]. Among myocardial stress biomarkers, sST2 has the strongest odds ratio to detect 30-day cardiovascular death and heart failure, even if compared with NT-proBNP and MR-proANP [22]. These studies used enzyme-linked immunoassay for sST2 measurement, which are completely different from our method of sST2 quantification. Similar to previous studies, our study also utilized thawed-frozen serum sample for sST2 measurement.

The practicality and simplicity of the POCT test in acute coronary syndrome is of paramount importance, because the fast result can rapidly guide the clinical decision. The enzyme-linked immunosorbent assay is a more sensitive method of measurement than POCT, however it is robust, time-consuming, and impractical causing its limited use in clinical daily practice. The ASPECT-PLUS ST2 test is a rapid quantitative lateral flow immunoassay for measurement of sST2 in human plasma. The fluorescent signal indicates interaction between antibodies against human sST2 which are tagged with a fluorescent dye [6]. The signal is measured with the ASPECT reader. The quantitative sST2 values are determined based on the linear calibration curve unique to each lot and are given as ng/mL [6]. The total duration from sample preparation to result interpretation is no more than 35 minutes which is sufficiently rapid. The promptness of POCT is its superiority over other test methods which are crucial factors in the setting of STEMI.

Several limitations were identified in our study. Firstly, the number of subjects was not large enough to support strong conclusions with sufficient statistical power for generalizability. Secondly, the blood samples used in this study were thawed-frozen samples which are not compatible in the real setting of acute disease, which preferably uses fresh samples. Last but not least was the uncertain determination of the cutoff value using the current POCT system because a significant amount of patients had the sST2 level beyond the detection range using the POCT.

In conclusion, the sST2 POCT measurement on admission was useful to identify patients at high risk to develop adverse cardiac events during acute intensive care of STEMI. Supramedian sST2 levels independently predicted adverse cardiac events during acute intensive care of STEMI.

## Figures and Tables

**Table 1:** The comparison of characteristics and sST2 level between controls and subjects with STEMI.

Characteristics	Controls, *n*=10	Subjects with STEMI, *n*=95	*p* value
*Demography*			
Age (years), mean ± SD	61.0 ± 2.9	58.2 ± 8.5	0.30
Male sex, *n* (%)	8 (80.0)	76 (80.0)	0.68
Body mass index, mean ± SD	26.3 ± 2.8	24.2 ± 2.8	0.06

*Risk factors*			
Diabetes mellitus, *n* (%)	5 (50.0)	28 (29.5)	0.18
Hypertension, *n* (%)	10 (100.0)	55 (57.9)	<0.01
Current smoking, *n* (%)	2 (20.0)	50 (52.6)	0.12

*Clinical parameters*			
Systolic b.p (mmHg), mean ± SD	132.0 ± 17.5	128.9 ± 26.4	0.72
Diastolic b.p (mmHg), mean ± SD	73.0 ± 9.7	78.9 ± 16.4	0.41
Heart rate (bpm), mean ± SD	73.0 ± 9.7	76.1 ± 18.5	0.60

*Laboratory examination*			
Hemoglobin (g/dL), mean ± SD	13.4 ± 1.8	13.8 ± 1.8	0.48
Leucocytes (10^3^/mm^3^), mean ± SD	8.3 ± 2.8	12.8 ± 3.2	<0.01
Platelets (10^3^/mm^3^), mean ± SD	283.5 ± 48.9	276.6 ± 97.7	0.83
Creatinine (mg/dL), mean ± SD	1.3 ± 0.4	1.3 ± 0.5	0.58
Glucose (mg/dL), mean ± SD	131.2 ± 41.1	181.5 ± 94.8	<0.01
sST2 (ng/mL), median (q1–q3)	28.5 (22.7–36.9)	152.1 (72.0–250.0)	<0.01^*∗*^

b.p = blood pressure; bpm = beat per minute; q1 = first quartile; q3 = third quartile; ^*∗*^Mann–Whitney *U* test.

**Table 2:** The comparison of characteristics between inframedian and supramedian sST2 in subjects with STEMI.

Characteristics	Inframedian sST2, *n*=48	Supramedian sST2, *n*=47	*p* value
*Demography*			
Age (years), mean ± SD	57.3 ± 8.7	59.1 ± 8.2	0.31
Male sex, *n* (%)	38 (79.2)	38 (80.9)	0.84
Body mass index, mean ± SD	24.3 ± 2.8	24.1 ± 3.0	0.79

*Risk factors*			
Diabetes mellitus, *n* (%)	16 (33.3)	12 (25.5)	0.41
Hypertension, *n* (%)	30 (62.5)	25 (53.2)	0.36
Current smoking, *n* (%)	24 (50.0)	26 (55.3)	0.60

*Clinical parameters*			
Onset (hour), mean ± SD	8.1 ± 6.9	7.4 ± 5.5	0.48
Systolic b.p (mmHg), mean ± SD	131.7 ± 27.9	126.0 ± 24.7	0.29
Diastolic b.p (mmHg), mean ± SD	80.3 ± 17.3	77.4 ± 15.4	0.39
Heart rate (bpm), mean ± SD	76.8 ± 17.1	75.4 ± 19.9	0.71
Killip II-IV, *n* (%)	3 (6.2)	5 (10.6)	0.44

*Laboratory examination*			
Hemoglobin (g/dL), mean ± SD	13.9 ± 1.8	13.6 ± 1.9	0.25
Leucocytes (10^3^/mm^3^), mean ± SD	12.9 ± 3.7	12.7 ± 2.8	0.76
Platelets (10^3^/mm^3^), mean ± SD	293.6 ± 107.8	259.3 ± 83.7	0.09
Creatinine (mg/dL), mean ± SD	1.2 ± 0.3	1.3 ± 0.7	0.09
Glucose (mg/dL), mean ± SD	184.9 ± 92.2	177.9 ± 98.4	0.72
Troponin I (ng/dL), mean ± SD	6.5 ± 10.5	9.4 ± 10.5	0.03^*∗*^
Creatine kinase-MB (IU), mean ± SD	93.2 ± 79.2	200.9 ± 204.3	<0.01^*∗*^
Total cholesterol (mg/dL), mean ± SD	189.2 ± 58.8	181.9 ± 43.7	0.50
LDL cholesterol (mg/dL), mean ± SD	123.9 ± 38.1	124.7 ± 35.7	0.91
HDL cholesterol (mg/dL), mean ± SD	43.8 ± 16.6	45.3 ± 8.9	0.59
Triglyceride (mg/dL), mean ± SD	134.7 ± 55.2	122.2 ± 75.8	0.36

*Infarct location*			
Anterior, *n* (%)	23 (47.9)	24 (51.1)	0.83
Inferior, *n* (%)	24 (50.0)	21 (44.7)	0.83

*Initial treatment*			
Primary PCI, *n* (%)	17 (35.4)	17 (36.2)	0.94
Fibrinolysis, *n* (%)	18 (37.5)	24 (51.1)	0.18
Heparinization, *n* (%)			0.96
LMWH	4 (8.3)	5 (10.6)	
UFH	37 (77.1)	36 (76.6)	
Fondaparinux	4 (8.3)	4 (8.5)	

b.p = blood pressure; bpm = beat per minute; LV = left ventricle; LDL = low-density liporotein; HDL = high-density lipoprotein; PCI = percutaneous coronary intervention; LMWH = low molecular weight heparin; UFH = unfractionated heparin; ^*∗*^Mann–Whitney *U* test.

**Table 3:** The mode of revascularization and time to revascularization between inframedian and supramedian sST2 in subjects with STEMI.

Mode of revascularization	Inframedian sST2, *n*=48	Supramedian sST2, *n*=47	*p* value
Primary PCI, *n*=34 (35.7%)	17 (35.4%)	17 (36.2%)	
Time to revascularization (minutes), mean ± SD	179.7 ± 40.3	156.4 ± 45.2	0.12
Fibrinolysis, *n*=42 (44.2%)	18 (42.9%)	24 (51.1%)	
Time to revascularization (minutes), mean ± SD	68.3 ± 26.3	65.6 ± 21.8	0.72
Failed fibrinolysis and rescue PCI, *n*=9 (9.5%)	2 (11.1)	7 (29.2)	0.15

PCI = percutaneous coronary intervention.

**Table 4:** Univariate and multivariable analysis of variables as predictors for adverse cardiac events.

Univariate analysis variables	OR (95% confidence interval)	*p* value
Increased age (>60 years)	1.09 (1.03–1.16)	0.005
Diabetes mellitus	2.69 (1.02–7.09)	0.046
Heart rate	1.02 (0.99–1.04)	0.178
Hemoglobin level	0.63 (0.46–0.86)	0.004
Leukocyte count	0.85 (0.71–1.02)	0.078
Creatinine level	1.91 (0.83–4.41)	0.131
Glucose level	1.01 (1.00–1.01)	0.071
Triglyceride level	0.99 (0.98–0.99)	0.015
Troponin I level	1.03 (0.99–1.08)	0.170
Anterior STEMI	2.58 (0.98–6.81)	0.055
Primary PCI	2.23 (0.87–5.73)	0.097
Supramedian sST2	4.35 (1.54–12.27)	0.006

Multivariable analysis variables	Adjusted OR (95% confidence interval)	*p* value

Increased age (>60 years)	1.14 (1.02–1.28)	0.021
Diabetes mellitus	2.31 (0.29–18.49)	0.430
Heart rate	1.04 (0.99–1.08)	0.070
Hemoglobin level	0.77 (0.48–1.23)	0.273
Leukocyte count	1.06 (0.76–1.48)	0.740
Creatinine level	2.03 (0.69–5.89)	0.194
Glucose level	1.00 (0.99–1.01)	0.965
Triglyceride level	0.96 (0.97–1.01)	0.078
Troponin I level	1.03 (0.96–1.11)	0.352
Anterior STEMI	4.73 (0.87–25.72)	0.072
Primary PCI	2.21 (0.45–10.74)	0.326
Supramedian sST2	6.27 (1.33–29.47)	0.020

## Data Availability

All authors take responsibility for all aspects of the reliability and freedom from bias of the data presented and their discussed interpretation.
